# Analysis of the Relationship Between Chemical Elements and Neural Necrosis Virus in Mugilids from the Southern Caspian Sea

**DOI:** 10.1007/s00244-025-01177-y

**Published:** 2026-01-20

**Authors:** Shima Bakhshalizadeh, Rafael Mora-Medina, Nahúm Ayala-Soldado

**Affiliations:** 1 Department of Marine Science, Caspian Sea Basin Research Center, University of Guilan, Rasht, Iran; 2 Department of Anatomy and Comparative Pathology and Toxicology, Faculty of Veterinary Medicine, University of Córdoba, Córdoba, Spain

## Abstract

For years, the Caspian Sea has been affected by chemical pollution resulting from human activities. More recently, Viral Nervous Necrosis (VNN) has emerged as a new threat, severely impacting fish populations in this aquatic ecosystem. The aim of this study was to evaluate differences between healthy and VNN-infected mullets along the southwestern coast of the Caspian Sea. A total of 63 individuals were randomly sampled, including 34 *Chelon auratus* and 29 *Chelon saliens*. Viral prevalence was higher in *C. saliens*. Statistical test revealed clear differences between healthy and infected individuals based on their elemental profiles. Infection with NNV was associated with significantly elevated concentrations of several metals, particularly Hg, Pb, Mo, V, and Cu, with Hg showing up to a tenfold increase in infected fish. These findings confirm that the southern Caspian Sea is contaminated with multiple trace elements, which not only compromise ecosystem health but may also predispose fish to viral infections such as VNN.

## Introduction

Chemical pollution—particularly from toxic metals—poses a major threat to aquatic ecosystems because of their toxicity, persistence, and capacity for bioaccumulation and biomagnification across food webs (Almafrachi et al. 2024; Naz et al. 2025). While metals can enter waters through natural weathering, contemporary evidence indicates that human-derived inputs—industrial discharges, agricultural runoff, and municipal/household wastewater—are the major contributors to elevated metal loads. These pressures coincide with seasonal shifts in water chemistry and measurable biological responses, with consistent links between physicochemical gradients, plankton community structure, and metal accumulation in aquatic biota (Ayub et al. 2024; Habib et al. 2024).

The Caspian Sea, situated at the crossroads of Europe and Asia, is the world’s largest enclosed water basin. This unique ecosystem hosts a remarkable biodiversity, including iconic species like sturgeons. However, due to its enclosed nature and limited water renewal capacity, the Caspian Sea is particularly vulnerable to chemical pollution. Over recent decades, intensified human activities, particularly oil extraction and coastal mining, have significantly elevated pollutant concentrations in this region (Lattuada et al. 2019). Among these contaminants, hydrocarbons are especially concerning due to their widespread release from extensive oil industry activities, negatively impacting critical components of the ecosystem such as microbial communities (Hassanshahian et al. 2012).

The Caspian Sea’s biodiversity includes numerous species uniquely adapted to its specific salinity and temperature conditions, making it an important hotspot for speciation and evolutionary processes (Karpinsky 2005). Particularly noteworthy are species within the family Mugilidae (mullets), which are economically valuable, abundant, and serve as effective bioindicators due to their sensitivity to environmental conditions (Çiloğlu 2023; Nită et al. 2022). Among these, *Chelon auratus* and *Chelon saliens* have proven particularly effective in indicating pollution levels in coastal marine environments (Grimmelpont et al. 2023; Bakhshalizadeh et al. 2022). Due to their demersal habits and dietary preferences, these fish readily accumulate metals such as copper (Cu), zinc (Zn), lead (Pb), and cadmium (Cd), potentially posing health risks to humans through seafood consumption (Habib et al. 2024).

Additionally, another critical threat to Caspian Sea fish populations, including mugilids, is the Nervous Necrosis Virus (NNV), a pathogen from the genus Betanodavirus that causes Viral Nervous Necrosis (VNN). This disease results in severe neurological damage and high mortality, particularly during the early developmental stages of fish. The virus’s ability to persist asymptomatically in carriers complicates efforts to control its spread, as seemingly healthy fish can unknowingly maintain and disseminate the virus within the aquatic environment (Chen et al. 2017; Tso and Lu 2018).

Considering the ecological, economic, and public health significance of *C. saliens* and *C. auratus*, this study aims to evaluate differences in chemical element accumulation between healthy fish and those infected with NNV along the southwestern coastline of the Caspian Sea. The outcomes of this research will provide crucial insights into the interaction between environmental contaminants and viral infections, supporting future environmental management and conservation strategies for this vital ecosystem.

## Materials and Methods

### Sample Site

**Fig. 1:**
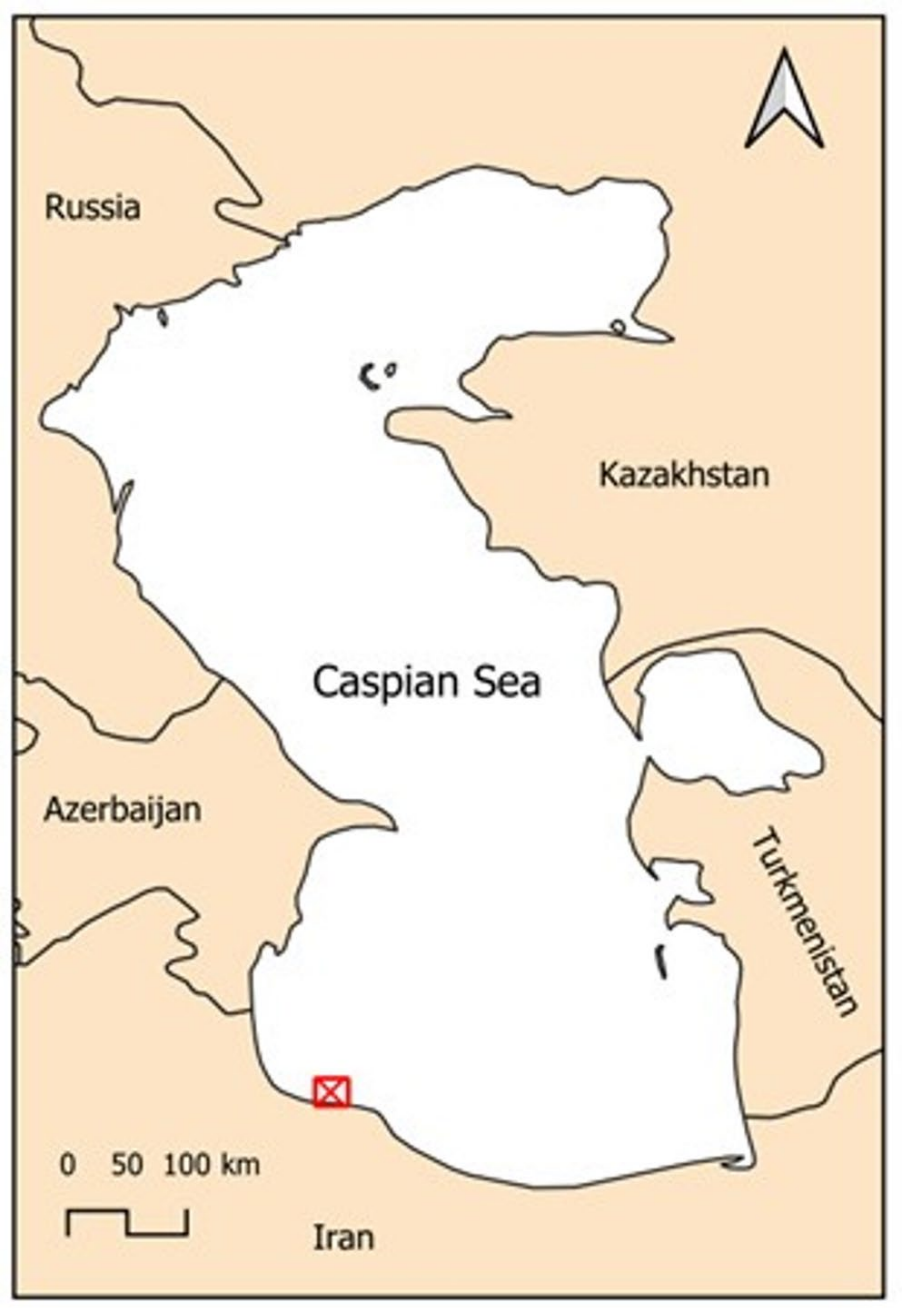
Localization of the sampling area (red rectangle) in the southern Caspian Sea

A total of 63 mullets, 34 *C. auratus* and 29 *C. saliens*, were randomly captured at a single coastal site on the southern Caspian Sea (Bandar Anzali, Iran), as shown in Fig. 1. All specimens were collected within one sampling campaign conducted during the legally open bony fish fishing season, with no stratification by site or time. Fish were captured with nets, euthanized by concussion, weighed, measured, aged, and kept on ice until tissue sampling.

Before collecting samples, the fish were externally cleaned with distilled water to remove dirt and contaminants. To determine whether the animals were positive for NNV, eye and brain samples were collected. For the eye, an incision was made in the ocular orbit and extracted using tweezers. For the brain, the skin and cranial bone were carefully removed with a scalpel and scissors, exposing the encephalon without causing damage. Subsequently, the skin in the caudal area was removed to obtain muscle tissue samples for chemical element analysis. Approximately 5 g of muscle was weighed using an analytical balance. All instruments were cleaned with a 1% nitric acid solution before each sampling. All samples were immediately frozen at -80 °C until analysis. The entire procedure was performed in accordance with Directive 2010/63/EU on the protection of animals used for scientific purposes and the ethical standards of the Research Ethics Committees of the University of Guilan (6958/P15; 2042024).

### Virus Determination

The determination of NNV was performed by RT-PCR following the protocol described by Soltani et al. (2010). Total RNA was extracted from brain and eye samples using the IQ2000 RNA extraction solution. Initially, 500 µL of RNA extraction solution containing phenol was added to 20 mg of tissue. The samples were homogenized and incubated at room temperature for 5 min. Subsequently, 100 µL of chloroform was added, vortexed for 20 s, and then centrifuged for 15 min. Approximately 200 µL of the upper aqueous phase was transferred to a new 0.5 mL tube containing 200 µL of isopropanol. The samples were then centrifuged at 12,000 × g for 10 min at 4 °C, after which the isopropanol was decanted. The RNA pellets were washed with 0.5 mL of 75% ethanol and centrifuged at 7,500 × g for 5 min at 4 °C to recover the RNA pellet. Ethanol was then decanted, and the dried pellet was dissolved in 200 µL of DEPC-treated water. RT-PCR assays were performed using the IQ2000 VNN detection kit (Farming Intelligence Tech. Corp., Taiwan). A total of 8 µL of the RT-PCR reaction mixture, containing 7.0 µL of premixed reagents, 0.5 µL of IQzyme, and 0.5 µL of reverse transcriptase enzyme, was mixed with 2 µL of extracted RNA sample or standard. The RT-PCR reaction was carried out at 42 °C for 30 min, followed by 94 °C for 2 min, and then 30 amplification cycles at 94 °C for 20 s, 62 °C for 20 s, and 72 °C for 30 s in a thermocycler (Bio-Rad). The final extension was performed at 72 °C for 30 s, followed by a hold at 20 °C for an additional 30 s.

After amplification, 5 µL of each RT-PCR product were resolved on a 2% agarose gel (1× TAE) alongside a 100-bp ladder, stained (SYBR Safe) and imaged (Gel Doc, Bio-Rad). Positive samples showed a single band at the expected size for the IQ2000 VNN assay (~ 289 bp). Representative amplicons were purified and bidirectionally Sanger-sequenced with the RT-PCR primers. Chromatograms were quality-checked, low-quality ends (QV < 20) were trimmed, and forward/reverse reads were assembled to a consensus. The consensus sequences were queried by BLASTn against GenBank; top hits corresponded to betanodavirus (NNV) with ≥ 99% identity and high query coverage, confirming target specificity.

Each RT-PCR batch included a kit positive control, a no-template control (NTC; nuclease-free water) and an extraction negative control (blank processed with the samples). Batches were accepted only when the positive control amplified at the expected size and both negative controls showed no amplification.

### Chemical Elements Analysis

Muscle tissue samples were subjected to wet digestion in triplicate. Approximately 1 g of fish tissue was digested with 10 mL HNO₃ (65%, Merck, Suprapur) in a microwave digestion system (MARS 6, CEM, USA). After complete digestion, samples were diluted to 25 mL with Milli-Q water and stored in polyethylene bottles until trace-element analysis. Determinations were performed by inductively coupled plasma mass spectrometry (ICP-MS, Agilent 7500 series), following the manufacturer’s recommendations for instrument optimization. Based on the Certified Reference Material DORM-4 (Fish Protein CRM, NRC Canada), the following elements were quantified: Ag, Al, As, Cd, Co, Cr, Cu, Fe, Pb, Li, Mn, Mo, Hg, Ni, Se, Sn, Sr, U, V, and Zn. Calibration and quality control were carried out using DORM-4 as a reference standard. Analytical recoveries ranged between 97 and 102%, and calibration curves were linear (r² > 0.994) for all elements. Blanks were processed identically to the samples, and concentrations were determined with standards prepared in the same acid matrix.

Method detection limits (MDL, 3σ) and limits of quantification (LOQ, 10σ) were established from procedural digestion blanks (reagents taken through the full microwave digestion and dilution) and converted to sample units considering the preparation (1 g tissue diluted to 25 mL). The resulting MDL/LOQ values (µg/kg, wet weight) were: Ag 0.75/2.50; Al 5.0/16.7; As 1.25/4.17; Cd 1.00/3.33; Co 0.50/1.67; Cr 0.50/1.67; Cu 0.75/2.50; Fe 15.0/50.0; Pb 0.75/2.50; Li 2.25/7.50; Mn 1.25/4.17; Mo 1.50/5.00; Hg 0.05/0.17; Ni 0.75/2.50; Se 0.75/2.50; Sn 1.25/4.17; Sr 0.75/2.50; U 0.75/2.50; V 0.75/2.50; Zn 1.50/5.00.

### Data Analysis

Data processing and statistical analyses were performed in R using the packages dplyr, tidyr, purrr, and broom for data handling, statistical testing, and visualization. First, the structure of the dataset was inspected, and the variables of interest were defined. The categorical variables species and NNV prevalence were converted into factors with predefined levels, and the numerical variables corresponding to the chemical elements were identified. For each element, descriptive statistics were calculated, and distribution normality was assessed with the Shapiro–Wilk test. Given the multifactorial design (species × NNV) and the lack of normality in most variables, a permutation-based ANOVA (aovperm function in R) was chosen as the most appropriate approach to evaluate main effects and interactions without assuming parametric distributions. Analyses were performed on concentrations transformed as log10(x + 1), using 5,000 permutations and the Freedman–Lane method, and *p-values* were obtained for the effects of species, NNV, and their interaction on each element. Results were considered statistically significant at *p* < 0.05.

In addition, a Principal Component Analysis (PCA) was conducted on the chemical element data, after log10(x + 1) transformation, centering, and scaling. The PCA was carried out using the base R function prcomp, and results were visualized with ggplot2, with variable labels added using ggrepel. The biplots included individual samples projected onto the first two principal components, loading vectors of the elements, and confidence ellipses summarizing group dispersion according to NNV prevalence.

## Results

The results regarding the identification of NNV were as follows: in the case of *C. auratus*, out of the 34 specimens analyzed, 23 fish tested positive. On the other hand, a higher prevalence was observed in *C. saliens*, where 23 samples tested positive and only 6 specimens were negative.

Regarding biological variables. negative *C. auratus* for NNV had a mean weight of 424 ± 154 g, a length of 32.4 ± 8.4 cm, and an age of 4.34 ± 0.78 years. On the other hand, negative *C. saliens* showed a mean weight of 245 ± 25.5 g, a length of 25.0 ± 14.7 cm, and an age of 3.33 ± 0.51 years. As for the positive specimens for NNV, *C. auratus* had a mean weight of 200 ± 83.78 g, a length of 30.5 ± 3.80 cm, and an age of 3.54 ± 0.52 years. Finally, positive *C. saliens* showed a mean weight of 113 ± 31 g, a length of 25.9 ± 2.2 cm, and an age of 3.74 ± 0.68 years.

Trace element concentrations (mg/kg wet weight) in muscle of *C. auratus* and *C. saliens* are shown as medians with interquartile ranges (Table 1). NNV prevalence was the factor associated with the largest number of significant differences. Concentrations of Ag, Al, Cd, Cr, Cu, Hg, Li, Mn, Mo, Ni, Pb, Se, U, and V were significantly higher in NNV-positive fish (*p* < 0.05) with particularly pronounced increases for Hg (≈ 10-fold). With respect to species effects, significant differences were detected for Cr (*p* = 0.008), Li (*p* = 0.011), Sn (*p* = 0.010), and Zn (*p* < 0.001). For instance, *C. saliens* showed higher concentrations of Cr and Zn, whereas *C. auratus* exhibited relatively higher levels of Sn. Species × NNV interactions were less common but reached significance for Li (*p* = 0.001), Mo (*p* = 0.007), and Sr (*p* = 0.047).

**Table 1:** Median concentrations [Q1–Q3] of trace elements (mg/kg wet weight) in muscle of *C. auratus* and *C. saliens*, separated by NNV prevalence (negative vs. positive). Results of univariate PERMANOVA are shown as p-values for the effects of species, NNV, and their interaction

Element (mg/kg)	Chelon auratus		Chelon saliens		*p*-value		
	NNV -	NNV+	NNV-	NNV+	Species	NNV	Interaction
Ag	0.001 [0.001–0.002]	0.768 [0.768–0.768]	0 [0–0.001]	0.734 [0.43–0.734]	0.153	< 0.001	0.147
Al	7.4 [6.55–11.35]	15.084 [14.512–40.991]	9.25 [6.825–11.675]	37.101 [13.256–74.037]	0.614	< 0.001	0.655
As	0.072 [0.072–0.072]	0.444 [0.357–0.631]	0 [0–0]	0.738 [0.478–1.381]	0.295	0.275	0.351
Cd	0.03 [0.017–0.053]	0.031 [0.016–0.062]	0.014 [0.01–0.024]	0.106 [0.032–0.223]	0.498	0.003	0.050
Co	0.68 [0.488–1.314]	0.859 [0.554–2.064]	0.977 [0.632–1.402]	1.599 [0.509–2.39]	0.570	0.208	0.905
Cr	1.071 [0.772–1.622]	1.462 [0.72–3.255]	2.196 [1.339–2.922]	4.783 [2.113–9.444]	0.008	0.025	0.255
Cu	1.933 [0.758–3.457]	7.018 [4.456–9.861]	3.03 [2.172–3.781]	7.321 [3.403–15.197]	0.466	< 0.001	0.615
Fe	34.875 [19.236–79.503]	28.175 [26.109–58.505]	40.967 [21.168–56.002]	69.096 [49.78–131.004]	0.185	0.091	0.131
Hg	0.05 [0.016–0.098]	0.462 [0.342–0.871]	0.025 [0.016–0.038]	0.508 [0.072–1.491]	0.615	< 0.001	0.914
Li	0.256 [0.217–0.483]	0.409 [0.234–0.688]	0.97 [0.951–1.31]	0.292 [0.241–0.513]	0.011	0.012	0.001
Mn	1.664 [0.935–2.88]	1.69 [0.944–4.267]	1.54 [0.782–2.766]	5.848 [2.893–10.176]	0.099	0.005	0.098
Mo	2.684 [1.988–15.397]	20.285 [13.288–35.949]	15.127 [7.063–28.879]	12.331 [9.23–23.641]	0.239	0.008	0.007
Ni	1.056 [0.41–1.547]	2.477 [1.559–4.152]	0.654 [0.496–0.717]	3.386 [1.995–5.846]	0.958	< 0.001	0.092
Pb	0.256 [0.087–0.407]	0.524 [0.256–0.787]	0.237 [0.165–0.295]	1.143 [0.485–1.726]	0.247	< 0.001	0.168
Se	0.263 [0.161–0.601]	0.591 [0.476–0.767]	0.41 [0.326–0.767]	0.742 [0.529–1.235]	0.127	0.005	0.992
Sn	0.668 [0.543–0.915]	0.232 [0.165–0.487]	0.847 [0.769–1.138]	1.744 [0.594–1.906]	0.010	0.635	0.140
Sr	0.407 [0.265–0.566]	1.029 [0.733–1.648]	0.939 [0.451–1.2]	0.634 [0.296–1.834]	0.670	0.103	0.047
U	0.045 [0.019–0.079]	0.112 [0.06–0.216]	0.025 [0.017–0.043]	0.067 [0.044–0.119]	0.236	0.006	0.236
V	0.627 [0.295–1.375]	6.365 [3.193–10.294]	0.743 [0.674–1.776]	4.506 [3.129–11.004]	0.562	< 0.001	0.865
Zn	13.095 [8.024–18.944]	18.587 [9.548–21.216]	42.385 [22.321–55.121]	29.565 [19.251–40.69]	< 0.001	0.725	0.527

**Fig. 2:**
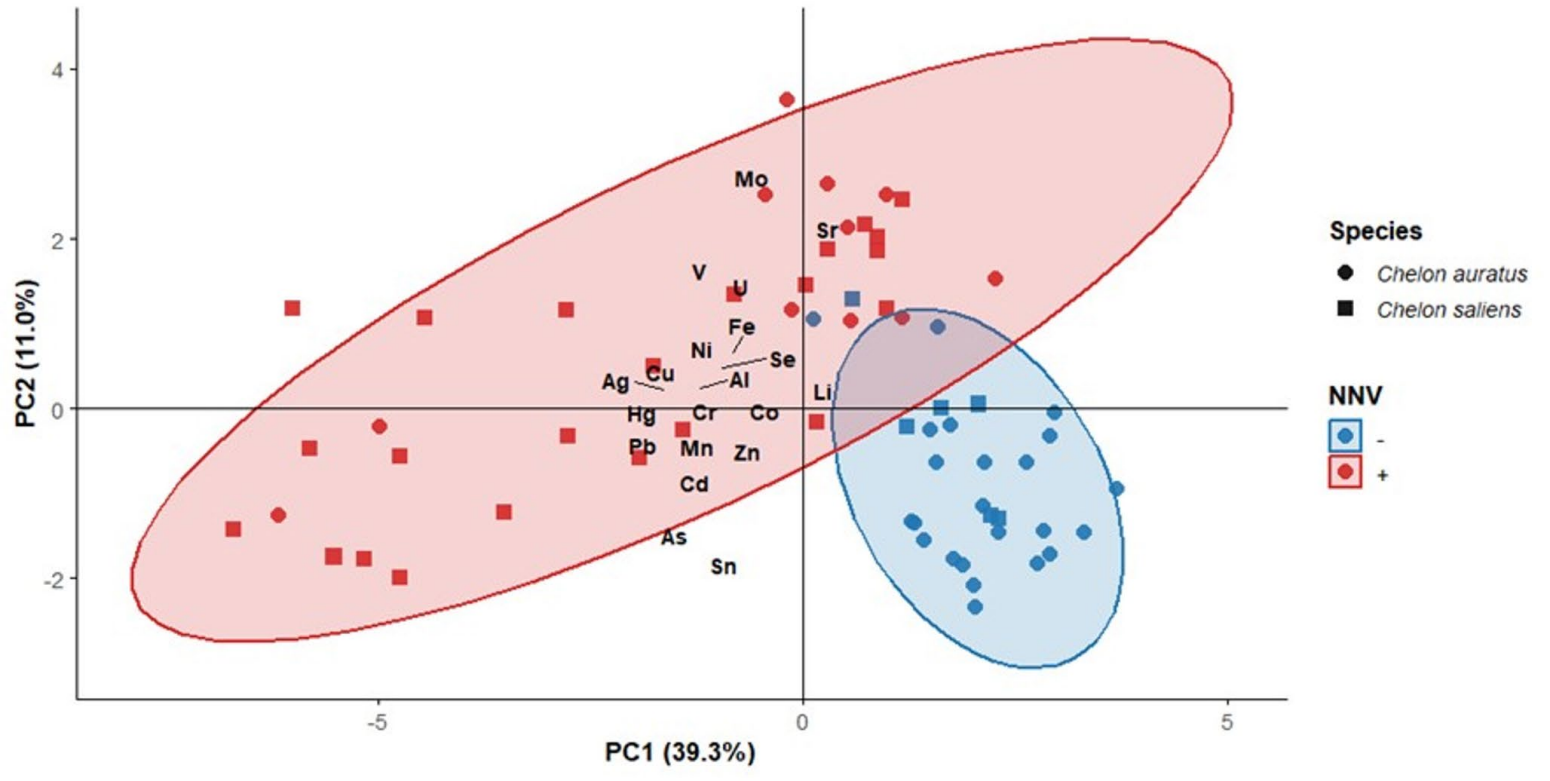
PCA biplot showing the distribution of *C. auratus* (circles) and *C. saliens* (squares) individuals according to the presence (NNV+) or absence (NNV–) of the NNV, with ellipses representing 95% confidence intervals

The PCA showed that the first two axes explained 50.3% of the total variability in elemental concentrations (PC1 = 39.3%, PC2 = 11.0%), while the remaining components each accounted for considerably smaller proportions of variance (< 7% each). The PCA biplot (Fig. 2) illustrates the distribution of individuals and the vectors of the elements with the highest loadings on each axis, along with confidence ellipses summarizing the variability associated with NNV prevalence. PC1 was mainly characterized by negative loadings for most elements, particularly Ag, Pb, Hg, Cd, and Cr (coefficients between − 0.29 and − 0.32), while Sr and Li showed loadings in the opposite direction. Along this axis, NNV-negative individuals of both species tended to have positive PC1 scores, indicating that the multielement variability captured by PC1 was primarily associated with differences in the accumulation of most chemical elements. PC2, in turn, was defined mainly by positive loadings of Mo (0.51), Sr (0.46), V (0.35), and U (0.31), together with negative loadings of As (–0.33) and Sn (–0.32). The distribution of individuals in the PC1–PC2 space (Fig. 2) revealed a clear separation according to NNV prevalence, whereas species differences were less pronounced. These results suggest that viral infection is associated with a bioaccumulation pattern characterized by simultaneous increases in several metals (particularly Cd, Hg, and Pb), while elements such as Mo and Sr contributed secondarily to the observed variability.

## Discussion

Previous environmental assessments from the southern Caspian Sea indicate persistently elevated background burdens of trace metals in coastal sediments and adjacent waters. Broad-scale surveys along Iran’s southern shoreline classify sediments as contaminated under standard geochemical indices, and studies of the southern Sefid-rud River—a major inflow—describe upstream-to-downstream increases and episodic ecological risk in deposited sediments (Abadi et al. 2019; Nakhaei et al. 2024). Collectively, these observations delineate a chronically exposed baseline against which our disease patterns should be interpreted.

Our findings show a strong association between NNV prevalence and elevated concentrations of several trace elements, including Hg, Pb, Mo, V and Cu, with additional contributions from Cd, Li, Se and U. The largest contrasts were observed for Hg, Mo and V, which differed markedly between infected and non-infected fish. This interpretation is consistent with established evidence that metal exposure can induce immunosuppression, oxidative stress and tissue alterations, thereby increasing infection risk and disease progression in aquatic organisms (Ali et al. 2019).

While some elements such as Fe, Zn, and Se are essential for normal physiology, their role is concentration dependent. Under normal environmental conditions, Fe and Zn are indispensable for enzymatic catalysis, immune function, and antioxidant defenses. However, at elevated concentrations they can promote the formation of reactive oxygen species (ROS), overwhelming natural antioxidant systems and increasing vulnerability to pathogens (Sfakianakis et al. 2015; Lushchak 2015). Selenium is particularly noteworthy because of its narrow threshold between essentiality and toxicity. At adequate concentrations it plays a protective role by activating antioxidant enzymes such as glutathione peroxidase (GPx), but even slight increases can significantly enhance oxidative stress, compromising fish health in a manner similar to other metals (Lall and Kaushik 2021).

The potential for synergistic interactions among trace elements must also be considered. For example, Hg and Se can exert antagonistic effects at low environmental concentrations, but combined exposure to elevated levels of both can amplify toxicity, impairing reproduction, embryonic development, and survival (Penglase et al. 2014). Metal-specific mechanisms linked to increased susceptibility to viral infections have also been documented. Pb, for instance, can impair immune function through MAPK signaling activation and miRNA-155 induction, leading to chronic inflammation in fish tissues (Jing et al. 2020). Similarly, Cu exposure can cause non-specific immune activation, reflected in the overexpression of heat shock proteins (HSP70 and HSP90) and immunoglobulin M (IgM) (Wang et al. 2019). Although initially protective, chronic overactivation of these stress responses may facilitate viral replication and interfere with adaptive immunity, thereby increasing susceptibility to viral pathogens such as NNV.

In general, elevated concentrations of trace elements, regardless of whether they are essential or non-essential, promote ROS overproduction and reduce antioxidant capacity. This imbalance drives oxidative stress and chronic inflammation, creating physiological conditions that favour viral replication and amplify pathological immune responses. Such cumulative impacts have major implications for fish health, resilience, and the sustainability of aquatic populations in contaminated environments.

In the southern Caspian basin, multiple anthropogenic sources, including industrial discharges, agricultural runoff, and oil and gas activities, have been implicated in sustaining metal inputs (Nematollahi et al. 2021; Hashempour-baltork et al. 2023). Contamination is evident not only in sediments and waters but also in wild fish, which frequently show higher burdens than aquaculture counterparts (Sobhanardakani 2017; Heshmati et al. 2017). Our recent work in the same region similarly identified elevated Pb and Cd in carnivorous species (Bakhshalizadeh et al. 2024), reinforcing the view that bioaccumulation mirrors the environmental signal. On a wet-weight basis, frequent exceedances occurred for Cd and Pb, with occasional exceedances for Hg. We compared values with the limits in Commission Regulation (EU) 2023/915. This underscores the urgent need to consider chemical pollution as a major cofactor in the emergence and severity of infectious diseases in aquatic ecosystems.

## Conclusion

Our results demonstrate that the presence of NNV in mullets is clearly associated with elevated concentrations of certain chemical elements, likely due to the creation of an environmentally unfavourable habitat resulting from contamination. Given the significant contamination previously reported in the southern Caspian Sea, particularly by metal(loid)s, our findings highlight the complexity and importance of interactions between chemical pollutants and emerging viral diseases in fish populations inhabiting polluted aquatic environments. This underscores the urgent need for interdisciplinary approaches that integrate ecotoxicology, environmental immunology, and aquatic organism health to effectively manage and mitigate these environmental threats in ecosystems such as the Caspian Sea.

## Data Availability

The data that support the findings of this study are available from the corresponding author.
